# Metabolic reprogramming by viruses in the sunlit and dark ocean

**DOI:** 10.1186/gb-2013-14-11-r123

**Published:** 2013-11-07

**Authors:** Bonnie L Hurwitz, Steven J Hallam, Matthew B Sullivan

**Affiliations:** 1 Ecology and Evolutionary Biology, University of Arizona, Tucson, AZ 85721, USA; 2 Department of Microbiology and Immunology, University of British Columbia, Vancouver, BC V6T 1Z4, Canada; 3 Graduate Program in Bioinformatics, University of British Columbia, Vancouver, BC V6T 1Z4, Canada; 4 Current address: Office of the Senior Vice President of Health Sciences, University of Arizona, Tucson, AZ 85724, USA

## Abstract

**Background:**

Marine ecosystem function is largely determined by matter and energy transformations mediated by microbial community interaction networks. Viral infection modulates network properties through mortality, gene transfer and metabolic reprogramming.

**Results:**

Here we explore the nature and extent of viral metabolic reprogramming throughout the Pacific Ocean depth continuum. We describe 35 marine viral gene families with potential to reprogram metabolic flux through central metabolic pathways recovered from Pacific Ocean waters. Four of these families have been previously reported but 31 are novel. These known and new carbon pathway auxiliary metabolic genes were recovered from a total of 22 viral metagenomes in which viral auxiliary metabolic genes were differentiated from low-level cellular DNA inputs based on small subunit ribosomal RNA gene content, taxonomy, fragment recruitment and genomic context information. Auxiliary metabolic gene distribution patterns reveal that marine viruses target overlapping, but relatively distinct pathways in sunlit and dark ocean waters to redirect host carbon flux towards energy production and viral genome replication under low nutrient, niche-differentiated conditions throughout the depth continuum.

**Conclusions:**

Given half of ocean microbes are infected by viruses at any given time, these findings of broad viral metabolic reprogramming suggest the need for renewed consideration of viruses in global ocean carbon models.

## Background

Marine ecosystems exert a profound influence on the operating conditions for life on earth [1,2], and their function is largely determined by matter and energy transformations flowing through microbial interaction networks [3,4]. Viral infection modulates these network properties through mortality, gene transfer, and metabolic reprogramming. In the case of metabolic reprogramming, bacterial viruses (phages) obtain genes from their hosts (termed auxiliary metabolic genes; AMGs) [5], and maintain them to bolster host metabolism during infection [5,6]. For example, cyanobacterial viruses (cyanophages) both harbor [7-10] and express [11,12] core photosynthesis genes that are modeled to improve phage fitness [13,14] and to influence the evolutionary trajectory of globally distributed host-encoded alleles [10,15].

Reactions of central metabolic pathways are strongly influenced by viral infection [16], because viral replication requires energy and materials for synthesis of macromolecules, including proteins, nucleic acids, and sometimes lipids. Emerging evidence supports a general model of viral reprogramming in which perturbations in glycolysis, the pentose phosphate pathway (PPP), and the tricarboxylic acid (TCA) cycle alter the metabolic flux and energy homeostasis of the host cell in support of viral replication and propagation at different stages of infection [17-19]. Environmental studies extend this concept to include cyanophages, as enhanced metabolic flux through the PPP increases production of NADH and ribose 5-phosphate, driving deoxynucleotide biosynthesis for phage replication [20].

Here we used the Pacific Ocean Virome (POV) dataset [21] to conservatively identify a sample subset suitable for quantitative AMG studies. This large and unique dataset has already enabled new estimates of the extent of the global virome three orders of magnitude less than previous estimates [22], and the discovery of the most abundant ocean viruses known (pelagiphages) [23]. Given that a highly purified [24-26], quantitative, viral metagenomic sample-to-sequence process [27,28] was used to prepare the POV dataset, and that it spans gradients of energy, nutrients, depth, and season throughout the Pacific Ocean, the POV dataset is ideal for ecological AMG studies. In fact, the purification process used here was estimated to be more than an order of magnitude better than other approaches to remove cellular bacterial contamination [26,29]. In the current study, we extensively documented trace cellular contamination in these highly pure POV data, then used 22 ‘ultra-clean’ viromes to map the nature and extent of metabolic reprogramming by ocean viruses, with an emphasis on AMGs modulating carbon flow through central metabolic pathways.

## Results and discussion

To develop a holistic perspective on carbon metabolism reprogramming potential, we analyzed the POV dataset spanning gradients of energy, nutrients, depth, and season. This dataset contains over 6 million reads and represents the first highly pure, nearly quantitative, pelagic ocean viromes (see Materials and methods for complete virome descriptions; see Additional file 1: Figure S1 for map) [21,26].

### Ruling out bacterial contamination in viromes

Given the need to differentiate between *bona fide* viral AMGs and low-level cellular DNA contamination, all viromes were prepared from prefiltered (<0.22 μm) seawater, so that the viral particles were concentrated before being purified by DNase and CsCl density gradients [26]. Although it is improbable that DNA would survive such processing without the protection of a protein capsid, it is not possible to exclude gene transfer agents (GTAs, which randomly package host DNA and co-purify with viral particles [30]) or cellular DNA contamination, without additional post-processing of genomic sequence information. In fact, sensitive kmer-based analysis using a smaller, previously available subset of these data (four viromes) showed that bacterial contamination was less than 0.002% (sample SFC.Spr.C.5m) in POV metagenomes, representing up to an order of magnitude improvement compared studies using other purification methods (sample STC.Spr.C.5m) [26,29]. Here we used multiple criteria for assessing GTAs and cellular DNA contamination in the POV dataset to identify a subset of viromes suitable for quantitative AMG studies. These analyses included small subunit (SSU) 16S ribosomal RNA (rRNA) gene content, taxonomy, fragment recruitment, and genomic context information. The findings are summarized in Table 1.

**Table 1:** **Pacific Ocean Virome viral samples used in searching for carbon metabolism genes**^**a**^

**Sample**	**16S + carbon metabolism genes in the same bacterial order**	**16S hits mostly toa single bacterial species**	**16S hits to many bacterial species**	**Random read recruitment to top bacterial genomes**	**Cellular contamination?**	**Reads, n**
Aphotic						
L.Win.O.1000m^b^	No	No	No	No	None	147,537
L.Win.O.2000m^b^	No	No	No	No	None	125,896
L.Spr.C.500m^b^	No	No	No	No	None	136,876
L.Spr.C.1300m^b^	No	No	No	No	None	98,478
L.Spr.I.1000m^b^	No	No	No	No	None	122,565
L.Spr.I.2000m^b^	No	No	No	No	None	49,914
L.Sum.O.1000m^b^	No	No	No	No	None	70,596
L.Spr.O.1000m^b^	No	No	No	No	None	101,179
L.Win.O.500m^b^	No	No	No	No	None	167,616
M.Fall.O.4300m^b^	No	No	No	No	None	144,588
L.Spr.C.1000m	Yes	Yes	No	Yes	High GTA^c^	97,126
L.Spr.I.500m	Yes	Yes	No	Yes	Low GTA^d^	58,108
L.Sum.O.500m	Yes	Yes	No	No	Low GTA^d^	42,118
L.Sum.O.2000m	Yes	No	No	No	Low GTA^d^	68,516
L.Spr.O.2000m	No	No	Yes	No	Low sporadic	55,332
M.Fall.O.1000m	No	No	No	Yes	Low GTA^d^	225,833
Photic						
L.Sum.O.10m^b^	No	No	No	No	None	165,256
L.Spr.C.10m^b^	No	No	No	No	None	107,244
L.Spr.I.10m^b^	No	No	No	No	None	92,415
L.Win.O.10m^b^	No	No	No	No	None	192,685
M.Fall.C.10m^b^	No	No	No	No	None	303,519
M.Fall.I.10m^b^	No	No	No	No	None	321,754
M.Fall.O.10m^b^	No	No	No	No	None	203,238
M.Fall.O.105m^b^	No	No	No	No	None	156,509
SFC.Spr.C.5m^b^	No	No	No	No	None	487,339
SFD.Spr.C.5m^b^	No	No	No	No	None	645,463
SFS.Spr.C.5m^b^	No	No	No	No	None	504,826
STC.Spr.C.5m^b^	No	No	No	No	None	821,404
GD.Spr.C.8m	Yes	Yes	No	Yes	Low GTA^d^	116,855
GF.Spr.C.9m	Yes	Yes	No	Yes	Low GTA^d^	82,739
L.Spr.O.10m	Yes	Yes	No	Yes	Low GTA^d^	75,036
M.Fall.I.42m	Yes	Yes	No	Yes	Low GTA^d^	31,528

^a^Samples were evaluated for gene transfer agents (GTAs) and sporadic contamination (see Results, section on ‘Ruling out bacterial contamination’) based on 16S analysis.

^b^Only samples indicated as having no contamination were used in the analysis.

^c,d^High GTAs are described as viromes with greater than 100 hits to a single bacterial species, low GTAs have less than 50 hits as determined by analysis (see Additional file 2: Figure S2).

^b^Sample metadata are further described by Hurwitz and Sullivan [21].

**Figure 1:**
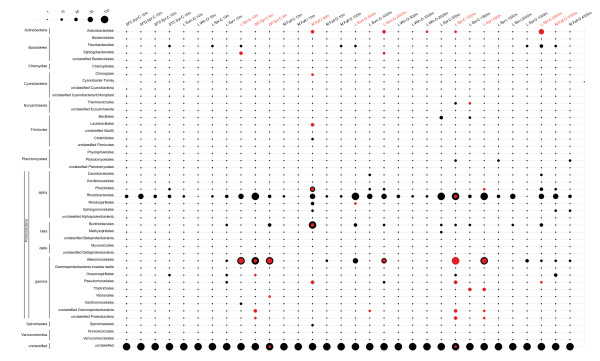
**Taxonomic distribution of viral metagenomic read hits to small subunit 16S ribosomal DNA and carbon metabolism genes by bacterial order.** 16S hits are noted in red and carbon metabolism gene hits are noted in black. Samples and metadata are further described by Hurwitz and Sullivan [21].

The 16S RNA gene is associated with cellular life forms, and its taxonomy typically correlates with functional gene content in microbial metagenomes [31]. As a first pass for cellular contamination in the POV dataset, these viromes were interrogated for 16S RNA gene content and taxonomy, and the findings compared with the taxonomy for identified (details below) carbon pathway AMGs (Figure 1). To be conservative in evaluating AMGs, the modest 16S RNA gene recovery from 9 of 32 POV viromes resulted in their exclusion from the study (Table 1; see Additional file 2: Figure S2). Notably, AMG taxonomy across all viromes partitioned between unassigned viromes and the *Rhodobacterales* within the *Alphaproteobacteria*, whereas 16S rRNA genes did not affiliate with this order in 23 viromes. We interpret this to suggest that the viruses infecting *Rhodobacterales* and/or the GTAs associated with them are prevalent components of Pacific Ocean waters. In most cases, carbon metabolism genes lacked taxonomy, and therefore these genes may be virally encoded, given that the viral world has been much less explored relative to the bacterial. A tenth sample, M. Fall.O.1000m, was subsequently excluded based on over-representation of protein-encoding genes (3.1%) from *Alcanivorax DG881* (see Additional file 3: Table S1), but lacked corresponding enrichment of 16S RNA genes affiliated with this order (Figure 1). We conservatively interpret this single taxon signal as a possible enrichment for GTAs, as cellular DNA contamination would lead to representation from a diversity of abundant taxa.

**Figure 2:**
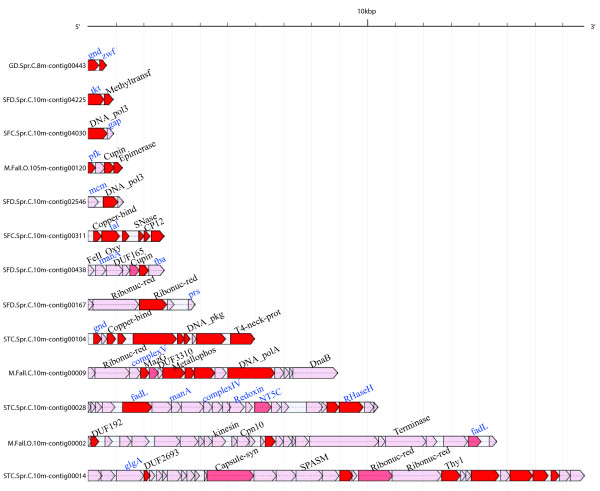
**Representative contigs containing carbon metabolism genes.** Example contigs containing carbon metabolism shown in blue, in context with other genes shown in black. Genes are colored based on superkingdom annotation: red, viral; light red, bacterial; pink, no superkingdom.

Next, genomic context or linkage information was used to validate viral AMG identification [32]. Although POV is the largest consistently prepared viral dataset available, the sequencing of any single virome remains shallow compared with more recent replicated Illumina sequenced viromes [33,34]. We found that in POV, only 17% of the contigs contained a gene with appropriate taxonomic annotation (superfamily designation), with most (87%) of these being only a single gene, while only 0.1% of all POV-derived contigs were relevant to our study in containing at least one carbon metabolism gene linked to a taxonomically informative annotation. Despite these limitations, 14 of the 35 carbon metabolism genes identified here, including 4 known (*fba*, *gnd*, *zwf*, *tal*) and 10 novel (*complex V*, *complex IV*, *fadL*, *gap*, *glgA*, *mcm*, *pfk*, *prs*, *tkt*, and *manA*), were validated as virus-encoded based on linkage information (all contigs, genes, and annotation are provided in GFF3 format in Additional file 4: Table S2). Several example contigs are shown in Figure 2 that have new carbon metabolism genes detected and their genomic context. Using these validated viral AMGs to mine available sequenced phage genomes (at NCBI as of June 2013), eight carbon metabolism genes were also identified in the genomes of several phage isolates (3 known and 5 new genes described below, Table 2).

**Table 2:** **List of validated carbon metaboli**sm **genes in known viral genomes**

**Carbon metabolism gene**	**Viral genome**	**Accession number**
Complex V	Environmental Halophage eHP-3	AFH21535.1
Complex V	Invertebrate iridescent virus 6	AAB94427.1
Complex V	*Salmonella* phage S16	AEO97118.1
*fadL*	*Aeromonas* phage PX29	ADQ52804.1
*fadL*	*Enterobacteria* phage K1E	CAJ29435.1
*fadL*	*Enterobacteria* phage SP6	AAP48767.1
*fadL*	*Enterobacteria* phage UAB_Phi78	ADW95239.1
*fadL*	*Enterobacteria* phage vB_EcoP_ACG-C91	AFH19857.1
*fadL*	*Persicivirga* phage P12024S	AFM54685.1
*fadL*	*Roseobacter* phage SIO1	AF189021_30
*glgA*	*Acanthocystis turfacea Chlorella* virus NE-JV-2	AGE56753.1
*glgA*	*Rhodothermus* phage RM378	NP_835600
*gnd*	Cyanophage Syn30	AGH56273.1
*gnd*	*Synechococcus* phage S-CAM8	AET72575.1
*gnd*	*Synechococcus* phage S-MbCM6	AFD02712.1
*gnd*	*Synechococcus* phage S-RSM4	CAR63315.1
*gnd*	*Synechococcus* phage S-SKS1	AGH31572.1
*gnd*	*Synechococcus* phage S-SM2	ADO97572.1
*gnd*	*Synechococcus* phage S-SSM5	ADO97956.1
*gnd*	*Synechococcus* phage syn9	ABX80643.1
*pfk*	*Prochlorococcus* phage P-SSM2	AAX44687.1
*pfk*	*Prochlorococcus* phage P-SSM2	ACY76185.1
*pfk*	*Synechococcus* phage S-CAM1	AGH26954.1
*tal*	Cyanophage MED4-213	AGH26225.1
*tal*	Cyanophage NATL1A-7	ADP00123.1
*tal*	Cyanophage S-TIM5	AEZ65636.1
*tal*	*Prochlorococcus* phage P-HM2	ADP00007.1
*tal*	*Prochlorococcus* phage P-SSM2	ACY76123.1
*tal*	*Prochlorococcus* phage Syn33	ADO99585.1
*tal*	*Synechococcus* phage S-RSM2	CAF32257.1
*tal*	*Synechococcus* phage S-RSM4	CAR63243.1
*tal*	*Synechococcus* phage S-SKS1	AGH31538.1
*tal*	*Synechococcus* phage S-SM2	ADO97591.1
*tkt*	*Micromonas pusilla* virus SP1	AET85010.1
*zwf*	*Synechococcus* phage S-CAM1	AGH26937.1
*zwf*	*Synechococcus* phage S-SM2	ADO97573.1

Gene abbreviations include: ATP synthase (complex V), long-chain fatty acid transporter (*fadL*), glycogen biosynthetic gene (*glgA*), 6-phosphogluconate dehydrogenase (*gnd*), 6-phosphofructokinase (*pfk*), transaldolase (*talC*), transketolase (*tkt*) and glucose 6-phosphate-1-dehydrogenase (*zwf*).

We next explored whether the gene signatures for carbon metabolism genes for the available paired viral and microbial metagenomes (the SIO viromes) [26] were similarly represented (Table 3). Although carbon metabolism genes were readily detectable in the viromes, they were at reduced (approximately one-fifth) abundance compared with the microbial metagenome. Further, and most compelling, was that only a subset (8 to 11 of the total of 54 genes analyzed; see Additional file 5: Table S3) of the carbon metabolism genes examined were detected in the viromes, whereas all were detected in the microbial metagenome. We interpret this to reflect a reduced universality of carbon metabolism genes in viruses compared with microbes. This parallels the ‘phage photosynthesis’ observations made in cyanophage genomes, showing that cyanosiphophages lack photosynthesis genes [35,36], and that many photosynthesis genes have sporadic patterns across cyanomyophage genomes [37] indicative of viral genome content leading to viral niche differentiation across varied hosts, environments, and infection styles.

**Table 3:** **Comparison of carbon metabolism genes detected in viral and microbial metagenomes**^**a**^

**Sample**	**Sample description**	**Carbon metabolism genes detected, n**^**b**^	**Normalized reads per carbon metabolism gene detected, n**^**c**^	**Carbon metabolism genes not detected, n**	**Normalized reads per carbon metabolism gene not detected, n**
SFC.Spr.C.5m	FeCl:CsCl + DNase	8	28 ± 23	46	1 ±1
SFD.Spr.C.5m	FeCl:DNase only	11	30 ± 27	43	2 ±2
SFS.Spr.C.5m	FeCl:Sucrose + DNase	11	25 ± 25	43	0 ±1
STC.Spr.C.5m	TFF:CsCl + DNase	17	23 ± 16	37	3 ±1
SM.Spr.C.5m	SIO microbial	54	161 ± 191	0	0 ±0

^a^Viromes were concentrated and purified using different protocols as described by Hurwitz *et al*. [26].

^**b**^Carbon metabolism genes (see Additional file 5: Table S3) were detected in viral and microbial metagenomes derived from the same water sample taken from Scripps Pier in San Diego, CA in April 2009.

^c^Carbon metabolism genes were considered to be detected if they had more than 5 normalized read hits.

Given these extensive efforts to identify contamination, we interpreted the remaining 22 viromes to be ‘ultra-clean’. In total, 35 carbon pathway AMGs remained identifiable out of 54 examined (see Additional file 5: Table S3) suggesting that they are *bona fide* viral AMGs*.* Although of course this is only a hypothesis until observed in fuller genomic context, the subset of central carbon metabolism genes and ecological gene distribution patterns observed in this study parallel confirmed findings in cyanophage genomes, and allude to a general paradigm of viral reprogramming of host metabolism in nature. The following scenarios provide plausible explanations of the biological roles of these genes in viruses.

### Carbon metabolism genes encoded by viruses in the sunlit photic ocean

**Figure 3:**
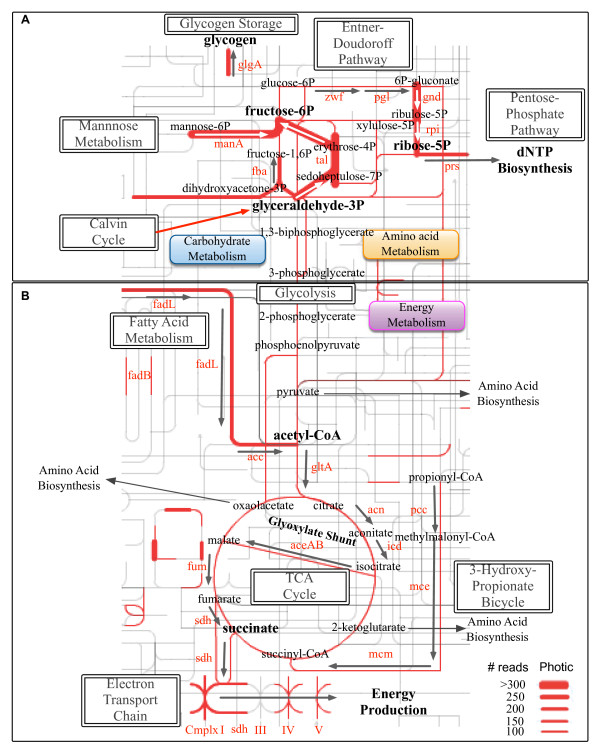
**Metabolic map of virus-encoded carbon metabolism host genes from 12 viromes in sunlit Pacific Ocean waters.** Red lines represent genes encoded in the photic zone. The width of the lines corresponds to the normalized read abundance as shown in the legend, and arrows correspond to the proposed flow through these pathways during viral infection. Enzymes are listed in red and compounds in black. **(A)** Virus-encoded host genes in glycolysis, fatty acid metabolism, the pentose phosphate pathway, and the Entner-Doudoroff pathway towards dNTP biosynthesis. **(B)** Virus-encoded host genes in glycolysis, fatty acid metabolism, the tricarboxylic acid (TCA) cycle, the electron transport chain, and components of the 3-hydroxypropionate Bicycle towards energy production. For map generation, see iPath [40].

In the sunlit photic ocean, carbon metabolism genes previously identified in cyanophage genomes (transaldolase (*talC*), glucose 6-phosphate-1-dehydrogenase (*zwf*), and 6-phosphogluconate dehydrogenase (*gnd*) in myoviruses, and *talC* in podoviruses [20,37,38]) and metagenomic surveys (fructose bisphosphate aldolase (*fba*) [32]) were recovered in POV datasets (Figure 3; see Additional file 5: Table S3). With regard to genes encoded in cyanophage genomes, Thompson and colleagues [20] proposed that during early infection, the Calvin cycle is inhibited via chloroplast protein-12 (*cp12*) to divert carbon towards the PPP by unidirectionally converting glyceraldehyde-3P (via *talC*) to fructose-6P. Fructose-6P can then produce reducing power (in PPP) and the carbon skeleton (ribose-5P) that phages need for dNTP biosynthesis via *zwf*, 6-phosphogluconolactonase (*pgl*), *gnd*, ribose-5-phosphate isomerase (*rpi*), and ribose-phosphate diphosphokinase (*prs*) (Figure 3). dNTP biosynthesis has been shown to be a bottleneck in phage replication [20,39]. The POV data supports and extends this proposition by including *rpi* and *prs*, two enzymes previously unobserved in viruses, as well as another carbon metabolism gene, mannose-6-phosphate isomerase (*manA*) (Figure 3; see Additional file 5: Table S3).

The *manA* gene was identified in all viromes at frequencies similar to the relatively ubiquitous cyanophage gene encoding the core photosystem II reaction center protein [10] (816 *manA* versus 3,379 *psbA* reads). In *Escherichia coli* K-12, ManA converts mannose-6P to fructose-6P for use in glycolysis [41]. Additionally, *talC*, previously observed in cyanobacterial T7-like podovirus and T4-like myovirus genomes [38], and expressed during cyanophage infection [42], was common in photic zone samples, presumably to convert glyceraldehyde-3P to fructose-6P. We posit that virus-encoded *manA* and *talC* allow diverse phages to utilize mannose and other glycolytic carbon sources for dNTP biosynthesis and reducing power (NADPH), using fructose-6P as a gateway to glucose-6P and PPP under low nutrient conditions. Interestingly, abundant POV-encoded PPP enzymes (for example, *gnd*, transketolase (*tkt*), and *talC*) (see Additional file 6: Table S4) represent all three enzymes whose metabolic flux is increased in starved *E. coli*[43]*.* Moreover, the glycogen biosynthetic gene (*glgA*), present in all viromes suggests that some viral infections trigger a starvation response in their hosts to redistribute carbon through non-glycolytic pathways [44,45].

Carbon metabolism genes may play a role in energy production (Figure 3). Identification of 6-phosphogluconate dehydratase (*edd*) and 2-keto-3-deoxy-6-phosphogluconate aldolase (*eda*) in the Entner-Doudoroff pathway (EDP) in photic samples is consistent with conversion of pyruvate to acetyl coenzyme A (acetyl-CoA) via pyruvate dehydrogenase complex subunits (*aceEF*) for use in energy production through the TCA cycle during viral infection (Figure 3).

Components of the TCA cycle including aconitase (*acn*), isocitrate dehydrogenase (*icd*), 2-oxoglutarate dehydrogenase (*sucABCD*), isocitrate lyase and glyoxylate shunt, and malate synthase A (*aceAB*) were identified in photic samples*.* In either the regular route through the TCA cycle or through the glyoxylate shunt, succinate offers a metabolic branch-point supporting either anapluerotic reactions or energy production. In the former, production of oxaloacetate supports pyrimidine catabolism and amino acid synthesis, while the latter can drive energy production through electron transport for phage replication. Consistent with this, genes encoding respiratory complex enzymes were identified in photic samples (Figure 3).

**Figure 4:**
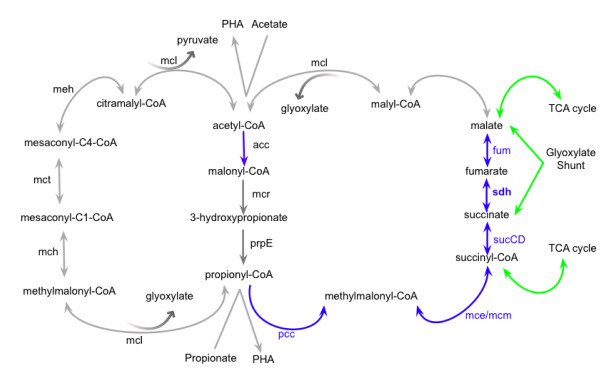
**Overview of Pacific Ocean Virome (POV)-encoded 3-hydroxypropionate Bicycle enzymes.** Enzyme names are listed as in Additional file 5: Table S3. The figure complements and highlights the pathways shown in Figures 3 and 5. The enzyme *acc* can also play a role in fatty acid metabolism.

In addition to genes involved in central metabolism, two new marine viral gene families encoding fatty acid metabolic subsystems were identified in photic samples (Figure 3). These include fatty acid oxidation complex (*fadB*), the long-chain fatty acid transporter (*fadL*), and components of the 3-hydroxypropionate (3HP) cycle (acetyl-CoA carboxylase (*acc*), propionyl-CoA carboxylase (*pcc*), methylmalonyl-CoA epimerase/mutase (*mcm*), *sucCD*, succinate dehydrogenase (*sdh*), fumarate hydratase (*fum*)). These observations are consistent with energy generation via fatty acid oxidation and balancing of TCA cycle intermediates during viral infection in the photic ocean. Redirecting carbon from fixation to energy production via *pcc*, *mce*, and *mcm* (Figure 4) may influence the carbon and nitrogen cycles through metabolically reprogramming the 3HP cycle for inorganic carbon fixation [46] in abundant marine *Crenarchaea*.

Viral gene families encoding central metabolic subsystems including glycolysis and pyruvate dehydrogenase were also detected, but to a lesser degree (Figure 3; see Additional file 6: Table S4). This is consistent with the hypothesis that viruses redirect carbon away from amino acid biosynthesis and use alternate pathways towards dNTP and energy production.

### Carbon metabolism genes encoded by viruses in the dark aphotic ocean

**Figure 5:**
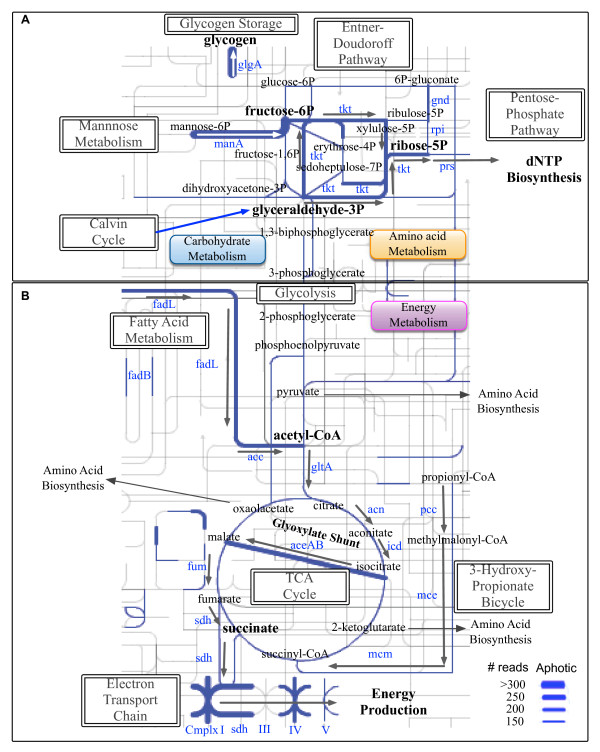
**Metabolic map of virus-encoded carbon metabolism host genes from 10 viromes in dark Pacific Ocean waters.** Blue lines represent genes encoded in the aphotic zone. The width of the lines corresponds to the normalized read abundance as shown in the legend and arrows correspond to the proposed flow through these pathways during viral infection. Enzymes are listed in blue and compounds in black. **(A)** Virus-encoded host genes in glycolysis, fatty acid metabolism, the pentose phosphate pathway, and the Entner-Doudoroff pathway towards dNTP biosynthesis. **(B)** Virus-encoded host genes in glycolysis, fatty acid metabolism, the tricarboxylic acid (TCA) cycle, the electron transport chain, and components of the 3-hydroxypropionate Bicycle towards energy production. For map generation, see iPath [40].

The dark aphotic ocean remains nearly completely unexplored for viruses, particularly for AMGs. In the deep Pacific Ocean pelagic waters, viral carbon metabolism gene families encoding subsystems including glycolysis, PPP, pyruvate dehydrogenase, EDP, the TCA cycle, and electron transport systems were detected (Figure 5; see Additional file 6: Table S4). Although similar in some senses to that in the photic zone, immediate and compelling contrasts between photic and aphotic zone samples were also observed. First, although aphotic and photic samples both have the potential to convert cellular mannose-6P to fructose-6P via *manA*, the subsequent conversion route of fructose-6P appears to differ between them (Figure 5). In aphotic samples, identification of transketolase (*tkt*) is consistent with the conversion of fructose-6P to erythrose-4P and xylulose-5P, which are both precursors for purine catabolism via ribose-5P and *prs*.

Second, abundant genes involved in fatty acid metabolism, the TCA cycle, and electron transport systems suggest that similar mechanisms for energy production in aphotic phage exist, as has already been described for photic phage [32], although more pronounced in the dark ocean as described below (Figure 5).

### Co-evolutionary niche differentiation between viruses in the sunlit and dark ocean

Comparison between photic and aphotic zone viromes showed niche differentiation consistent with either phototrophic or chemotrophic host metabolisms (Figure 3, Figure 5; see Additional file 6: Table S4). Photic zone viromes were enriched for gene families encoding pathways related to dNTP or reducing power (for example, PPP) with carbon and energy probably coming from photosynthetic AMGs (for example, *psbA*) and to a lesser degree through fatty acid metabolism and energy production in the TCA cycle and electron transport chain. By contrast, aphotic zone viromes were enriched for gene families encoding energy conversion pathways (for example, *glgA*, *fadL*, and four electron transport chain enzymes). Further, a greater abundance of *glgA* in aphotic viromes suggests that ‘starving’ the host by removing glucose might be a fundamental first step in phage energy production, whereby *aceAB* is activated in nutrient-limited conditions. initiating the glyoxylate shunt towards increased energy production and decreased amino acid biosynthesis (Figures 3 and 5).

## Conclusions

Viral lysis alone is responsible for the largest carbon flux in the oceans, calculated as 150 gigatons per year [47] without including the surface ocean virus-mediated photosynthesis that appears from microbial metagenomic surveys to be considerable [48]. Here we show that virus-encoded carbon metabolism genes go well beyond photosynthesis and photic ocean viral communities, in ways that probably differentially influence microbial-driven carbon metabolism in both the sunlit and dark ocean. It is likely that no single virus harbors all AMGs in this reprogramming repertoire, but instead that AMGs are maintained in rate-limiting steps specific to particular virus-host infection pairs. Given that microbial metabolic fluxes are tuned to environmental conditions [49], similar tuning for virus-encoded AMGs as described here across sunlit and dark ocean niches is not surprising. Further, recent studies highlight just how much remains unknown about the types of viruses that exist in nature [23,50-52], so it should also be no surprise that a ubiquitous viral AMG signal, so central to modulating carbon metabolism outputs, might have gone undetected.

Together, these data are consistent with widespread viral modulation of microbial interaction networks in the marine environment spanning multiple ecological scales, from global carbon pumps to metabolite flux within and between cells [4]. These iterative shunting effects indicate the essential role of viruses in shaping ecological patterns and biogeochemical processes through information exchange and metabolic reprogramming. Phenotypically, viral upregulation of key metabolic enzymes compensates for imbalances arising during infection, commonly through shortcut pathways associated with stressed cells. Such metabolic reprogramming between infected and non-infected microbial cells critically alters cellular carbon flux, which, has major implications for understanding nutrient and energy flow in the earth system, given that half of marine bacteria are infected by viruses at any given time [53]. The challenge now is to combine ‘gene ecology’-style surveys with emerging and yet to be -developed technologies [54-56] and theory [57-59], in order to more fully map what infects what in the genomic context, which is necessary to more comprehensively understand and model the metabolic reprogramming capabilities of viruses.

## Materials and methods

We have made the protocols, scripts and associated documentation available online [60,61].

### Virome preparation and sequencing

Viromes used in this study were taken from the POV dataset [21], with the exception of one virome (L. Spr.C.1000m) that was thought to contain GTAs. Briefly, viromes were derived from four geographic regions in the Pacific Ocean: 1) Scripps Pier in San Diego, CA, USA (SIO), 2) Line 67 in Monterey, CA (MBARI), 3) LineP in the Eastern Subarctic Northern Pacific (LineP), and 4) the Great Barrier Reef in Australia (GBR) (see Additional file 1: Figure S1). Four SIO viromes were derived from a single coastal seawater sample (depth of 5 meters) collected in spring of 2009, but concentrated and purified using different protocols. Seven MBARI viromes were derived from three stations at multiple depths in fall 2009 (coastal station H3 (10 meters); intermediate/upwelling station 67 to 70 (10, 42 meters); open ocean station 67 to 155 (10, 105, 1000, and 4300 meters). MBARI viromes at depths 42 meters (stations 67 to 70), and 105 meters (station 67 to 155) are from the deep chlorophyll maximum (DCM). Eighteen LineP viromes were derived from three stations at variable depths and seasons (coastal station P4 (spring: 10, 500, and 1300 meters); intermediate station P12 (spring: 10, 500, 1000, and 2000 meters); open ocean station P26 (spring 10, 1000, and 2000 meters; fall: 10, 500, 1000, and 2000 meters; and winter: 10, 500, 1000 and 2000 meters). Depths for LineP viromes represented gradients in oxygen concentration on the transect including above (500 meters), within (1000 meters), and below (2000 meters) the oxygen minimum zone. Two GBR viromes were derived from coastal reef surface samples near Dunk (8 meters) and Fitzroy (9 meters) Islands.

Viromes were prepared from 31 separate virus communities (as described above) using a 1.6 μm Whatman GF/A grade glass microfiber filter followed by a 0.22 μm filter to prefilter the seawater, after which particles were concentrated by FeCl precipitation [25], and purified by DNase and CsCl [26]. DNA was then extracted from purified particles using Wizard PCR DNA Purification Resin and Minicolumns [62], and randomly sheared and amplified using a modified linker amplification (LA) protocol [24,62]. LA DNA was sequenced using about a quarter-plate of GS FLX Titanium sequencing chemistry on a 454 Genome Sequencer [63] per virome, and the resulting reads were quality filtered to remove reads with ambiguous bases or those that differed by more than two standard deviations from the mean length and quality score [21,26]. The resulting approximately 6 M read POV dataset is freely available at Community Cyberinfrastructure for Advanced Microbial Ecology Research and Analysis (CAMERA) [64] as projects CAM_P_0000914 and CAM_P_0000915, at metaVIR [21], and by personal request from the authors. The data are also available at the iPlant Collaborative [65]. To access the POV dataset, login to iPlant, navigate to the discovery environment, open the data window, and browse to the community directory:(imicrobe/pov).

Protein clusters were generated from the data as described previously [21]. Briefly, each virome was assembled by binning reads by their k-mer frequency, and assembling each bin using Velvet version 1.0.15 (hash length = 29, -long) [21], Open reading frames (ORFs) were predicted using Prodigal (in metagenomics mode) on contigs and singleton reads [66], and ORFs were then used to generate protein clusters using cd-hit version 4.5.5 [67] from POV, the Global Ocean Survey [68], and all available viral proteins in Genbank (including 33,857 proteins) as of June 2011. All protein clusters and their annotation are available at iPlant in the community directory (imicrobe/pov).

### Taxonomic and functional classifications

Taxonomy and function were assigned to virome-derived ORFs by comparison (BLASTX, E value < 0.001) against the Similarity Matrix of Proteins (SIMAP, 25 June 2011 release; [69] using a custom pipeline (blastpipeline_simap.tar). SIMAP is a comprehensive and consolidated protein data set derived from Genbank, PDB, RefSeq, SwissProt, and Trembl, which provides pre-computed protein domains and annotation, thereby facilitating computation. Briefly, top hits to SIMAP entries were used for taxonomy assignments at the species, family, and genus level based on the NCBI taxonomic lineage, and for functional annotation using SIMAP data from the Gene Ontology, Pfam, Tigrfam, and PIR databases, as well as non-SIMAP data from Eggnog [70], PhAnToMe [71] and ACLAME [72]. Bacterial metagenomes were similarly annotated, except that BLASTN [73] was used to compare against the SIMAP database.

### Mapping reads to protein clusters and quantifying hits to carbon metabolism genes

To maximize annotation, protein clusters were leveraged to assign annotation to reads, with the idea that individual reads may lack annotation but the protein cluster may be assigned to a function of interest. To do this, protein clusters were identified that matched a list of curated carbon metabolism TIGRFAM/Pfams (see Additional file 5: Table S3). Reads were then mapped to ORFs in protein clusters using BLASTX [73] (E value < 0.001) and inherited the functional annotation of the top match associated with ORFs in that protein cluster. This approach allowed us to double the number of reads we found associated with carbon metabolism genes (6,733 reads using read based annotation, and 12,423 reads using protein cluster annotation). Read counts to each carbon metabolism gene were determined by summing up sequencing effort-weighted read counts by sample (see Additional file 6: Table S4). Read counts were weighted by dividing the number of reads by the total nucleotides for that sample, and multiplying by the average number of nucleotides for all samples.

### Ruling out bacterial contamination

#### 16S ribosomal DNA analysis

Viral metagenomic reads were assigned to SSU 16S rRNA using top BLASTN hits against release 10_30 from the Ribosomal Database Project (RDP) [74]. The top hits were required to have 75% coverage for the shortest read and 97% identity. Taxonomy data for bacterial order was derived from the definition line associated with the top hit from the Ribosomal Database Project. Taxonomic data for bacterial order for each of the carbon metabolism read hits were taken from the SIMAP hit as described above.

#### Finding contigs containing both carbon metabolism genes and known viral genes

Reads for each of the POV samples were assembled using newbler version 2.5.3 using default parameters. ORFs were found on all newbler contigs using Prodigal version 2.5.0 in metagenomic mode (-meta). ORFs were compared with SIMAP (as of 20 June 20 2013) using BLASTP as described above for functional and taxonomic annotation. ORFs matching carbon metabolism genes were found on contigs as noted above, and passed through a secondary filter to search for ORFs on the same contig matching the superfamily ‘Viruses’ based on SIMAP annotation. Contigs that contained a single ORF designated as both a carbon metabolism gene and of viral origin were retained (that is, carbon metabolism genes found on viral genomes), in addition to contigs that contained at least one carbon metabolism gene and one gene of known viral origin. Newbler contigs are available at iPlant in the community directory (imicrobe/pov).

#### Additional files

The following additional data are available with the online version of this paper. Additional file 1 is a figure (Figure S1) showing the sample collection sites for the POV dataset. Additional file 2 is a figure (Figure S2) showing a comparison of small subunit 16S ribosomal DNA viral metagenomic read hits to all species of bacteria versus a single top bacterial species. Additional file 3 is a table (Table S1) listing the percentage of bacterial proteins in the top five bacterial species with virome hits. Additional file 4 is a table (Table S2) listing contigs, genes, and annotation in GFF3 format for all contigs containing at least one carbon metabolism gene (as defined in Table S3) and at least one gene of viral origin. Additional file 5 is a table (Table S3) listing central carbon metabolism genes analyzed. Additional file 6 is a table (Table S4) listing read abundances for genes in Table S3 for each POV metagenome.

## Abbreviations

3HP: 3-hydroxypropionyl; AMG: Auxiliary metabolic gene; CoA: Coenzyme A; EDP: Entner-Doudoroff pathway; GTA: Gene transfer agent; LA: Linker amplification; ORF: open reading frame; POV: Pacific Ocean virome; PPP: Pentose phosphate pathway; SIMAP: Similarity Matrix of Proteins; TCA: Tricarboxylic acid cycle.

## Competing interests

The authors declare that they have no competing interests.

## Authors’ contributions

BLH and MBS conceived the study; BLH wrote the code and analyzed data; all three authors wrote the paper. All authors read and approved the final manuscript.

## Authors’ information

All metagenomic sequences were deposited to CAMERA [64] under the following project accessions: CAM_P_0000914 and CAM_P_0000915. Metagenomic sequences, assemblies, and annotation are available at iPlant [65] in the community directory (imicrobe/pov). Correspondence and requests for materials should be addressed to mbsulli@email.arizona.edu.

## Supplementary Material

Additional file 1: Figure S1 Map of viromes from the Pacific Ocean Virome (POV) dataset included in this study. **(A)** A global map showing all POV sampling sites; **(B)** sampling sites for viromes from Great Barrier Reef (GBR), Australia; **(C)** sampling sites for viromes from Monterey Bay, CA (MBARI); **(D)** sampling sites for viromes from Scripps Pier, San Diego, CA (SIO); **(E)** sampling sites for viromes from LineP, Eastern Subarctic Northern Pacific. All viromes that were designated as having sporadic or Gene transfer agent (GTA) contamination are noted in red. Click here for file

Additional file 2: Figure S2 Comparison of small subunit 16S ribosomal DNA virome read hits to all species of bacteria versus a single most abundant bacterial species. Click here for file

Additional file 3: Table S1 Percentage of proteins hit in genomes for the top five bacterial species in viromes. Sample SMS.Spr.C.5 m is a microbial sample that is included for comparison. Click here for file

Additional file 4: Table S2 GFF3 formatted file of Pacific Ocean Virome (POV) contigs containing carbon metabolism genes and at least one gene of viral origin. Click here for file

Additional file 5: Table S3 Description of central carbon metabolism genes used in analyses. Read abundances are summarized here and further documented in Table S4. Abbreviations for pathways are as follows: PPP, pentose phosphate pathway; EDP, Entner-Doudoroff Pathway; 3-HP, 3-hydroxypropionate Bicycle; ETC., electron transport chain; TCA, tricarboxylic acid cycle; FM, fatty acid metabolism. Genes that have been noted in viral genomes or metagenomes are denoted by ‘yes’ in the previously documented column. Click here for file

Additional file 6: Table S4 Sequencing effort-weighted read abundance in viromes for genes shown in Table S3. Column headers are: gly, glycolysis; edp, Entner–Doudoroff pathway; ppp, pentose phosphate pathway; nuc, dNTP biosynthesis; 3 hp, 3-hydroxypropionyl bicycle; ps, photosynthesis etc., electron transport chain; tca, TCA cycle; fa, fatty acid metabolism. Further information for each sample is provided in Table 1 and Hurwitz *et al*. [21]. Click here for file
