# Supplementary figures and images for: Metabolic reprogramming by viruses in the sunlit and dark ocean
Source: Genome Biol. 2013 Nov 7;14(11):R123. doi: 10.1186/gb-2013-14-11-r123 (PMC4053976; doi:10.1186/gb-2013-14-11-r123)

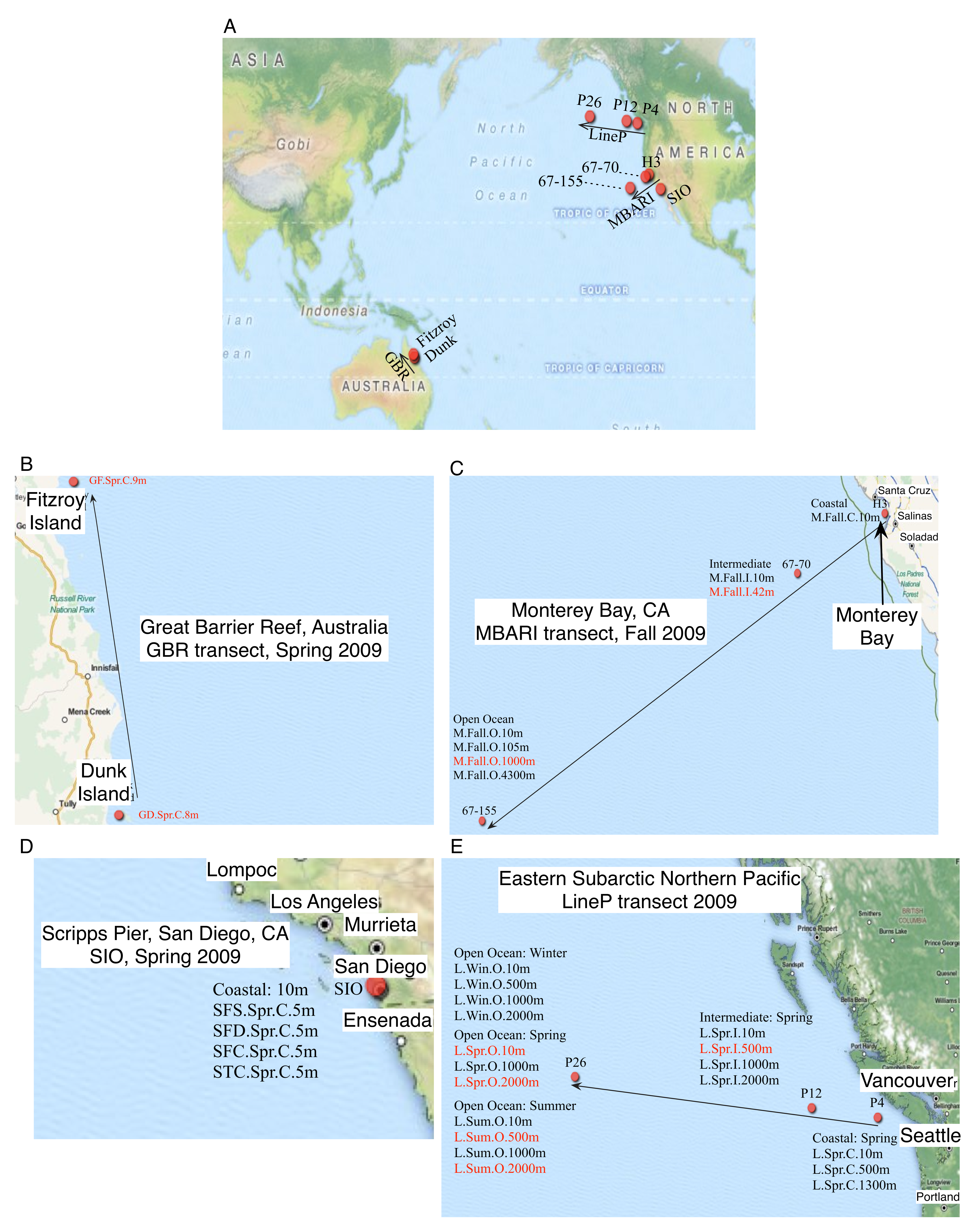

Supplement: Additional file 1: Figure S1 — Map of viromes from the Pacific Ocean Virome (POV) dataset included in this study. (A) A global map showing all POV sampling sites; (B) sampling sites for viromes from Great Barrier Reef (GBR), Australia; (C) sampling sites for viromes from Monterey Bay, CA (MBARI); (D) sampling sites for viromes from Scripps Pier, San Diego, CA (SIO); (E) sampling sites for viromes from LineP, Eastern Subarctic Northern Pacific. All viromes that were designated as having sporadic or Gene transfer agent (GTA) contamination are noted in red. [file gb-2013-14-11-r123-S1.png]

**A**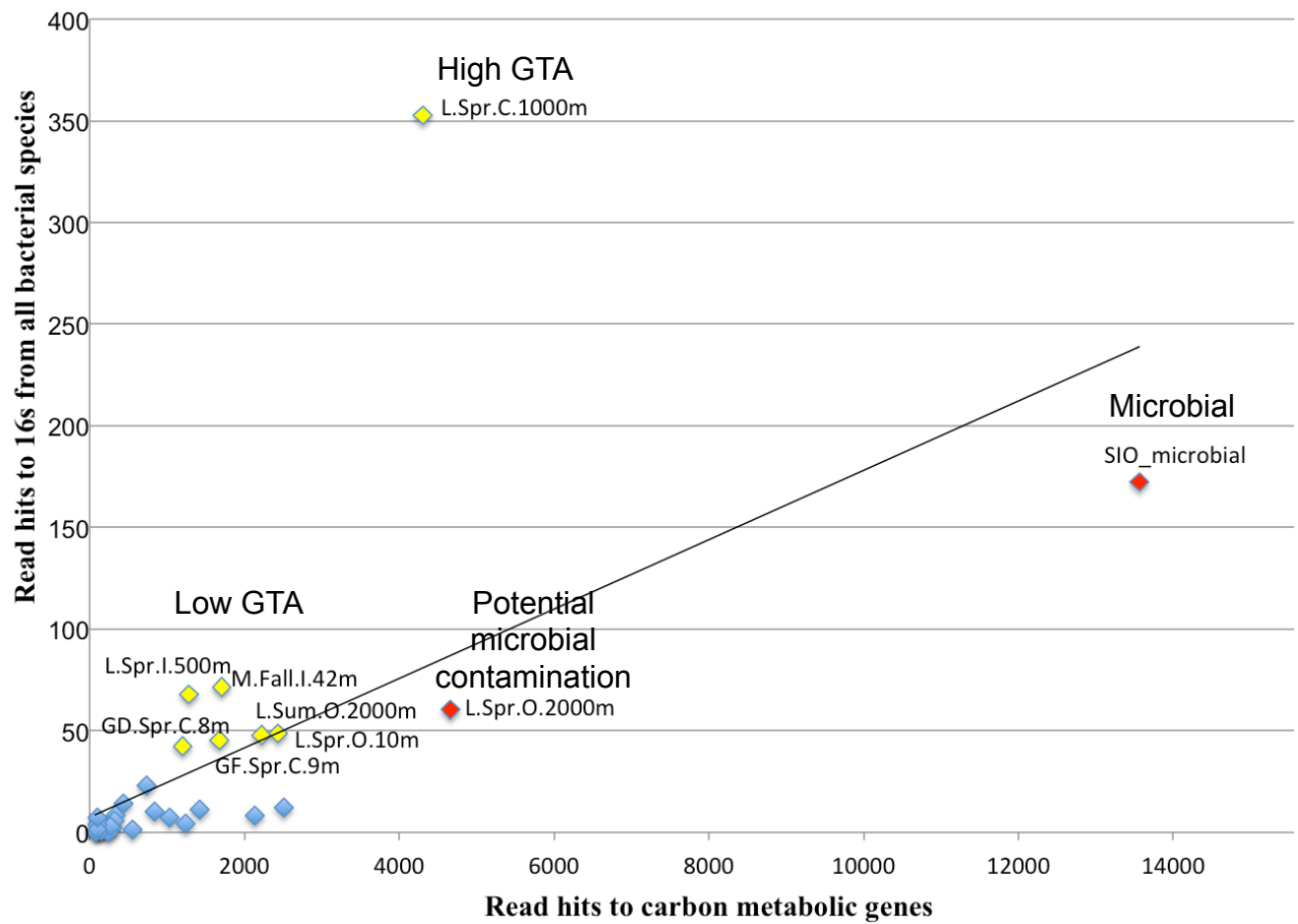**B**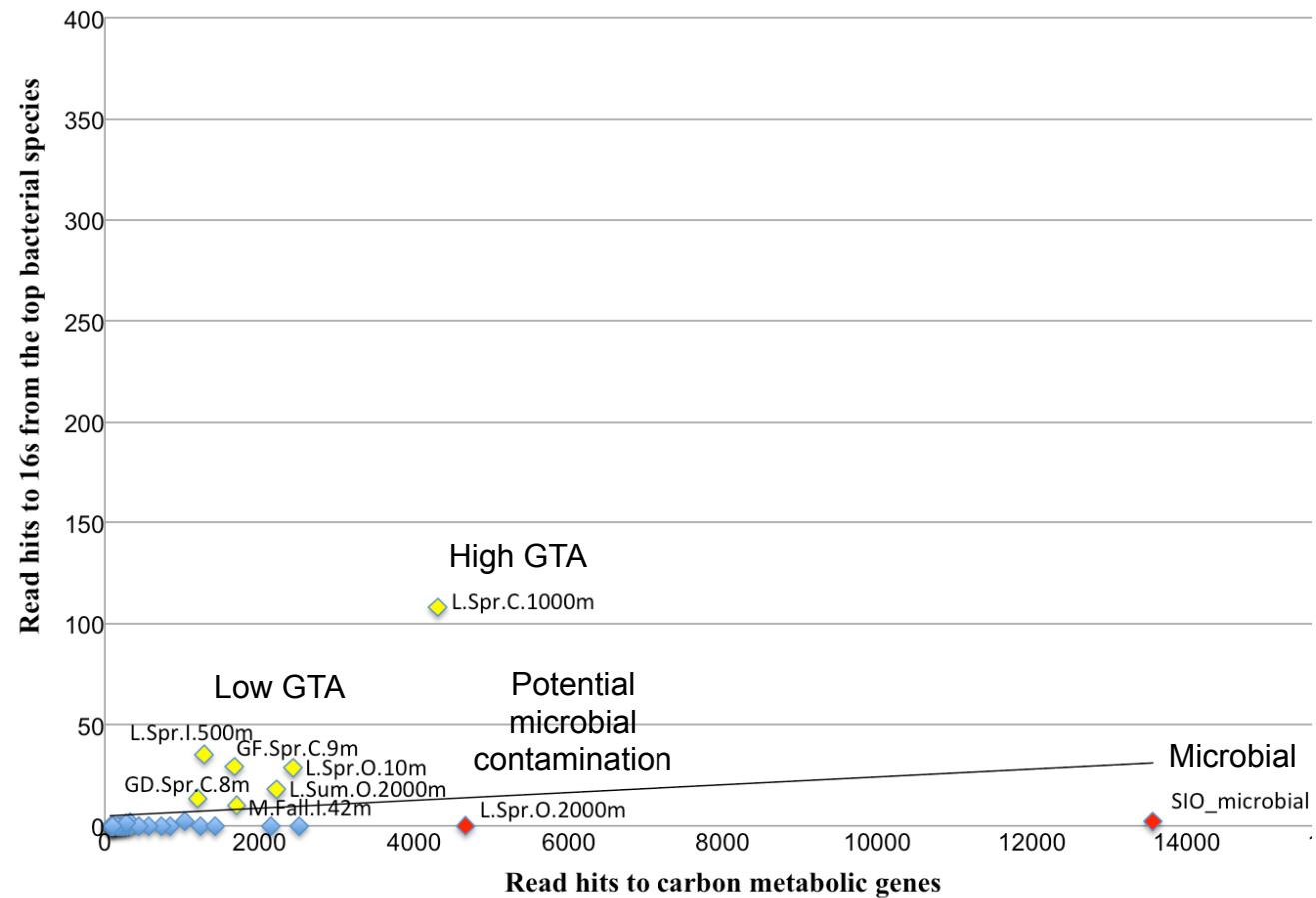

Supplement: Additional file 2: Figure S2 — Comparison of small subunit 16S ribosomal DNA virome read hits to all species of bacteria versus a single most abundant bacterial species. [file gb-2013-14-11-r123-S2.pdf]
