# Supplementary material for: Metabolic reprogramming by viruses in the sunlit and dark ocean
Source: Genome Biol. 2013 Nov 7;14(11):R123. doi: 10.1186/gb-2013-14-11-r123 (PMC4053976; doi:10.1186/gb-2013-14-11-r123)
Supplement: Additional file 3: Table S1 — Percentage of proteins hit in genomes for the top five bacterial species in viromes. Sample SMS.Spr.C.5 m is a microbial sample that is included for comparison. [file gb-2013-14-11-r123-S3.docx]

**Table S1. Percentage of proteins hit in genomes for the top 5 bacterial species in viral metagenomes.** Sample SMS.Spr.C.5m is a microbial sample that is included for comparison.

| **Sample** | **Top bacterial species** | **% Proteins hit (top1 bacteria)** | **Average % proteins hit (other top 4 bacteria)** |
| --- | --- | --- | --- |
| SMS.Spr.C.5m | *Alpha proteobacterium HIMB114* | 5.08% | 0.02% |
| STC.Spr.C.5m | *Acidobacterium sp. SP1PR4* | 0.13% | 0.43% |
| L.Spr.C.1000m | *Alteromonadales bacterium TW-7* | 12.95% | 0.16% |
| GD.Spr.C.8m | *Alteromonadales bacterium TW-7* | 7.73% | 0.28% |
| L.Spr.O.10m | *Alteromonadales bacterium TW-7* | 7.22% | 0.35% |
| GF.Spr.C.9m | *Alteromonadales bacterium TW-7* | 6.17% | 0.57% |
| L.Spr.I.500m | *Alteromonadales bacterium TW-7* | 2.51% | 0.15% |
| M.Fall.O.1000m | *Alcanivorax sp. DG881* | 3.10% | 0.17% |
| M.Fall.I.42m | *Burkholderia sp. 383* | 0.97% | 0.20% |
| L.Sum.O.2000m | *Alteromonadales bacterium TW-7* | 0.67% | 0.08% |
| M.Fall.O.4300m | *Alcanivorax sp. DG881* | 0.66% | 0.06% |
| L.Spr.C.500m | *Alteromonadales bacterium TW-7* | 0.54% | 0.02% |
| L.Sum.O.500m | *Burkholderia sp. 383* | 0.49% | 0.08% |
| L.Spr.I.10m | *Alteromonadales bacterium TW-7* | 0.35% | 0.07% |
| M.Fall.I.10m | *Alcanivorax sp. DG881* | 0.30% | 0.04% |
| L.Spr.O.2000m | *Burkholderia sp. 383* | 0.27% | 0.09% |
| L.Spr.C.1300m | *Alcanivorax sp. DG881* | 0.27% | 0.08% |
| M.Fall.C.10m | *Alcanivorax sp. DG881* | 0.25% | 0.03% |
| L.Spr.O.1000m | *Alteromonadales bacterium TW-7* | 0.22% | 0.33% |
| L.Spr.I.2000m | *Ralstonia sp. 5747FAA* | 0.21% | 0.16% |
| M.Fall.O.10m | *Alcanivorax sp. DG881* | 0.21% | 0.03% |
| L.Sum.O.1000m | *Burkholderia sp. 383* | 0.18% | 0.03% |
| L.Win.O.10m | *Alpha proteobacterium HIMB114* | 0.14% | 0.02% |
| L.Sum.O.10m | *Alpha proteobacterium HIMB114* | 0.14% | 0.08% |
| L.Spr.C.10m | *Alteromonadales_bacterium_TW-7* | 0.13% | 0.08% |
| L.Spr.I.1000m | *Alpha proteobacterium HIMB114* | 0.11% | 0.09% |
| M.Fall.O.105m | *Alteromonadales bacterium TW-7* | 0.08% | 0.02% |
| L.Win.O.2000m | *Bradyrhizobium sp. BTAi1* | 0.07% | 0.02% |
| L.Win.O.1000m | *Alteromonadales bacterium TW-7* | 0.07% | 0.02% |
| L.Win.O.500m | *Alteromonadales bacterium TW-7* | 0.07% | 0.03% |
| SFD.Spr.C.5m | *Acidobacterium sp. SP1PR4* | 0.06% | 0.01% |
| SFS.Spr.C.5m | *Acidobacterium sp. SP1PR4* | 0.04% | 0.19% |
| SFC.Spr.C.5m | *Acidobacterium sp. SP1PR4* | 0.02% | 0.02% |
