# Supplementary material for: Metabolic reprogramming by viruses in the sunlit and dark ocean
Source: Genome Biol. 2013 Nov 7;14(11):R123. doi: 10.1186/gb-2013-14-11-r123 (PMC4053976; doi:10.1186/gb-2013-14-11-r123)
Supplement: Additional file 5: Table S3 — Description of central carbon metabolism genes used in analyses. Read abundances are summarized here and further documented in Table S4. Abbreviations for pathways are as follows: PPP, pentose phosphate pathway; EDP, Entner-Doudoroff Pathway; 3-HP, 3-hydroxypropionate Bicycle; ETC., electron transport chain; TCA, tricarboxylic acid cycle; FM, fatty acid metabolism. Genes that have been noted in viral genomes or metagenomes are denoted by ‘yes’ in the previously documented column. [file gb-2013-14-11-r123-S5.docx]

|  |  |  |  |  |  |  |  |  |  |
| --- | --- | --- | --- | --- | --- | --- | --- | --- | --- |

**Table S3. Description of central carbon metabolic genes analyzed in POV viral metagenomes.** Read abundances are summarized here and further documented in Table S4. Abbreviations for pathways are as follows: PPP:Pentose Phostphate Pathway, EDP:Entner Doudoroff Pathway, 3-HP:3-Hydroxypropionate Bicycle, ETC:Electron Transport Chain, TCA:TCA cycle and FA:Fatty acid metabolism. Genes that have been discovered in viral genomes or metagenomes are denoted by “yes” in the previously documented column.

| **Pathway** | **Gene** | **Gene description** | **EC #** | **Reaction** | **Previously documented** | **Photic > #reads** | **Aphotic > #reads** |
| --- | --- | --- | --- | --- | --- | --- | --- |
| starch synthesis | *glgA* | glycogen synthase | E2.4.1.21 | ADP-glucose + Amylose <=> ADP + Amylose | no | 250 | 300 |
| starch synthesis | *glgC* | glucose-1-phosphate adenylyltransferase | E2.7.7.27 | ATP + D-Glucose 1-phosphate <=> Diphosphate + ADP-glucose | no | 1 | 1 |
| starch synthesis | *pgm* | phosphoglucomutase | E5.4.2.2 | D-Glucose 1-phosphate <=> D-Glucose 6-phosphate | no | 50 | 200 |
| glycolysis | *pgi* | phosphoglucose isomerase | E5.3.1.9 | D-Glucose 6-phosphate <=> D-Fructose 6-phosphate | no | 1 | 3 |
| fructose mannose metabolism | *manA* | mannose-6-phosphate isomerase | E5.3.1.8 | D-Mannose 6-phosphate <=> beta-D-Fructose 6-phosphate | no | 300 | 300 |
| glycolysis | *pfk* | 6-phosphofructosekinae | E2.7.1.11 | ATP + D-Fructose 6-phosphate <=> ADP + D-Fructose 1,6-bisphosphate | no | 1 | 1 |
| glycolysis | *fba* | fructose bisphosphate aldolase | E4.1.2.13 | D-Fructose 1,6-bisphosphate <=> Glycerone phosphate + D-Glyceraldehyde 3-phosphate | yes | 200 | 50 |
| glycolysis | *tpi* | triose phosphate isomerase | E5.3.1.1 | D-Glyceraldehyde 3-phosphate <=> Glycerone phosphate | no | 1 | 1 |
| glycolysis | *gap* | glyceraldehyde 3-phosphate dehydrogenase | E1.2.7.6 | D-Glyceraldehyde 3-phosphate + H2O + 2 Oxidized ferredoxin <=> 3-Phospho-D-glycerate + 2 H+ + 2 Reduced ferredoxin | no | 100 | 150 |
| glycolysis | *pgk* | phosphoglycerate kinase | E2.7.2.3 | ATP + 3-Phospho-D-glycerate <=> ADP + 3-Phospho-D-glyceroyl phosphate | no | 1 | 1 |
| glycolysis | *gpm* | 2,3-bisphosphoglycerate-dependent phosphoglycerate mutase | E5.4.2.1 | 2-Phospho-D-glycerate <=> 3-Phospho-D-glycerate | no | 1 | 1 |
| glycolysis | *eno* | enolase | E4.2.1.11 | 2-Phospho-D-glycerate <=> Phosphoenolpyruvate + H2O | no | 1 | 50 |
| glycolysis | *ppsA* | phosphoenolpyruvate synthetase | E6.3.2.36 | ATP + (R)-4-Phosphopantoate + beta-Alanine <=> AMP + Diphosphate + D-4'-Phosphopantothenate | no | 1 | 50 |
| glycolysis | *pyk* | pyruvate kinase | E2.7.1.40 | ATP + Pyruvate <=> ADP + Phosphoenolpyruvate | no | 1 | 50 |
| glycolysis | *ppc* | phosphoenolpyruvate carboxylase | E4.1.1.31 | Orthophosphate + Oxaloacetate <=> H2O + Phosphoenolpyruvate + CO2 | no | 1 | 1 |
| glycolysis | *pckA* | phosphoenolpyruvate carboxykinase | E4.1.1.49 | ATP + Oxaloacetate <=> ADP + Phosphoenolpyruvate + CO2 | no | 1 | 50 |
| PPP | *gnd* | 6-phosphogluconate dehydrogenase | E1.1.1.44 | 6-Phospho-D-gluconate + NADP+ <=> D-Ribulose 5-phosphate + CO2 + NADPH + H+ | yes | 300 | 200 |
| PPP | *rpi* | ribose-5-phosphate isomerase | E5.3.1.6 | D-Ribulose 5-phosphate <=> D-Ribose 5-phosphate | no | 300 | 150 |
| dNTP biosynthesis | *prs* | ribose-phosphate diphosphokinase | E2.7.6.1 | ATP + D-Ribose 5-phosphate <=> AMP + 5-Phospho-alpha-D-ribose 1-diphosphate | no | 150 | 50 |
| PPP | *rpe* | ribulose-5-phosphate 3-epimerase | E5.1.3.1 | D-Ribulose 5-phosphate <=> D-Xylulose 5-phosphate | no | 1 | 1 |
| PPP | *tkt* | transketolase | E2.2.1.1 | D-Fructose 6-phosphate + D-Glyceraldehyde 3-phosphate <=> D-Erythrose 4-phosphate + D-Xylulose 5-phosphate | no | 150 | 300 |
| PPP | *tal* | transaldolase | E2.2.1.2 | Sedoheptulose 7-phosphate + D-Glyceraldehyde 3-phosphate <=> D-Erythrose 4-phosphate + D-Fructose 6-phosphate | yes | 300 | 50 |
| EDP | *zwf* | glucose 6-phosphate-1-dehydrogenase | E1.1.1.49 | D-Glucose 6-phosphate + NADP+ <=> D-Glucono-1,5-lactone 6-phosphate + NADPH + H+ | yes | 100 | 1 |
| EDP | *pgl* | 6-phosphogluconolactonase | E3.1.1.31 | D-Glucono-1,5-lactone 6-phosphate + H2O <=> 6-Phospho-D-gluconate | no | 1 | 1 |
| EDP | *edd* | phosphogluconate dehydratase | E4.2.1.12 | 6-Phospho-D-gluconate <=> 2-Dehydro-3-deoxy-6-phospho-D-gluconate + H2O | no | 50 | 100 |
| EDP | *eda* | oxaloacetate decarboxylase | E4.1.2.14 | 2-Dehydro-3-deoxy-6-phospho-D-gluconate <=> D-Glyceraldehyde 3-phosphate + Pyruvate | no | 50 | 50 |
| 3-HP | *pcc* | Propionyl-CoA carboxylase | E6.4.1.3 | ATP + Propanoyl-CoA + HCO3- <=> ADP + Orthophosphate + (S)-Methylmalonyl-CoA | no | 1 | 150 |
| 3-HP | *mce* | Methylmalonyl-CoA epimerase | E5.1.99.1 | (R)-Methylmalonyl-CoA <=> (S)-Methylmalonyl-CoA | no | 1 | 1 |
| 3-HP | *mcm* | Methylmalonyl-CoA mutase | E5.4.99.2 | (R)-Methylmalonyl-CoA <=> Succinyl-CoA | no | 1 | 50 |
| ECE | *complexI* | NADH dehydrogenase | E1.6.5.3 | Ubiquinone + NADH + H+ <=> Ubiquinol + NAD+ | yes | 300 | 300 |
| ECE | *complexIII* | Ubiquinol cytochrome C reductase | E1.10.2.2 | Ubiquinol + 2 Ferricytochrome c <=> Ubiquinone + 2 Ferrocytochrome c + 2 H+ | no | 100 | 100 |
| ECE | *complexIV* | Cytochome C oxidase | E1.9.3.1 | Oxygen + 4 Ferrocytochrome c2 <=> 4 Ferricytochrome c2 + 2 H2O | no | 150 | 300 |
| ECE | *complexV* | ATPase | E3.6.3.14 | ATP + H2O <=> ADP + Orthophosphate | no | 50 | 250 |
| Pyruvate synthesis | *aceEF* | pyruvate dehydrogenase | E1.2.1.51 | Pyruvate + CoA + NADP+ <=> Acetyl-CoA + CO2 + NADPH + H+ | no | 50 | 200 |
| TCA | *gltA* | citrate synthase | E2.3.3.8 | ADP + Orthophosphate + Acetyl-CoA + Oxaloacetate <=> ATP + Citrate + CoA | no | 1 | 50 |
| TCA | *acn* | aconitase | E4.2.1.3 | Citrate <=> Isocitrate | no | 100 | 250 |
| TCA | *icd* | isocitrate dehydrogenase | E1.1.1.41 | Isocitrate + NAD+ <=> 2-Oxoglutarate + CO2 + NADH + H+ | no | 1 | 50 |
| TCA | *aceA* | isocitrate lyase | E4.1.3.1 | Isocitrate <=> Succinate + Glyoxylate | no | 100 | 250 |
| TCA | *sucAB* | 2-oxoglurarate dehydrogenase | E1.2.4.2 | 2-Oxoglutarate + CoA + NAD+ <=> Succinyl-CoA + CO2 + NADH + H+ | no | 50 | 250 |
| TCA | *aceB* | malate synthase | E2.3.3.9 | Acetyl-CoA + H2O + Glyoxylate <=> (S)-Malate + CoA | no | 100 | 250 |
| TCA | *sucCD* | Succinyl-CoA synthetase | E6.2.1.5 | ADP + Orthophosphate + Succinyl-CoA <=> ATP + Succinate + CoA | no | 1 | 50 |
| TCA | *sdh* | succinate dehydrogenaase | E1.3.99.1 | Succinate + FAD <=> FADH2 + Fumarate | no | 100 | 200 |
| TCA | *fum* | fumarase | E4.2.1.2 | Fumarate + H2O <=> (S)-Malate | no | 1 | 100 |
| TCA | *mdh* | malate dehydrogenase | E1.1.1.37 | (S)-Malate + NAD+ <=> Oxaloacetate + NADH + H+ | no | 1 | 50 |
| 3-HP | *acc* | Acetyl-CoA carboxylase | E6.4.1.2 | ADP + Orthophosphate + Malonyl-CoA <=> ATP + Acetyl-CoA + HCO3- | no | 50 | 100 |
| FA | *fadL* | long-chain fatty acid outer membrane porin | E2.3.1.- | fatty acid degradation | no | 200 | 300 |
| FA | *fadB* | fatty acid oxidation complex | E4.2.1.17 | (3S)-3-Hydroxyacyl-CoA <=> trans-2,3-Dehydroacyl-CoA + H2O | no | 50 | 1 |
| 3-HP | *prpE* | propionyl-CoA synthase | E6.2.1.17 | Propionyladenylate + CoA <=> AMP + Propanoyl-CoA, ATP + Propanoate + CoA <=> AMP + Diphosphate + Propanoyl-CoA | no | 0 | 0 |
| 3-HP | *mcl* | malyl-CoA lyase/beta-methylmalyl-CoA lyase | E4.1.3.24 | (3S)-Citramalyl-CoA <=> Acetyl-CoA + Pyruvate, (3S)-3-Carboxy-3-hydroxypropanoyl-CoA <=> Acetyl-CoA + Glyoxylate | no | 0 | 0 |
| 3-HP | *mcr* | malonyl-CoA reductase | E1.2.1.75 | 3-Oxopropanoate + CoA + NADP+ <=> Malonyl-CoA + NADPH + H+ | no | 0 | 0 |
| 3-HP | *mch* | mesaconyl-C1-CoA hydratase | none | L-erythro-3-Methylmalyl-CoA <=> Mesaconyl-CoA + H2O | no | 0 | 0 |
| 3-HP | *mct* | mesaconyl-CoA-C1-C4 CoA transferase | none | Mesaconyl-CoA <=> Mesaconyl-C4-CoA | no | 0 | 0 |
| 3-HP | *meh* | mesaconyl-C4-CoA hydratase | none | Mesaconyl-C4-CoA + H2O <=> (3S)-Citramalyl-CoA | no | 0 | 0 |
