# Supplementary material for: Metabolic reprogramming by viruses in the sunlit and dark ocean
Source: Genome Biol. 2013 Nov 7;14(11):R123. doi: 10.1186/gb-2013-14-11-r123 (PMC4053976; doi:10.1186/gb-2013-14-11-r123)
Supplement: Additional file 6: Table S4 — Sequencing effort-weighted read abundance in viromes for genes shown in Table S3. Column headers are: gly, glycolysis; edp, Entner–Doudoroff pathway; ppp, pentose phosphate pathway; nuc, dNTP biosynthesis; 3 hp, 3-hydroxypropionyl bicycle; ps, photosynthesis etc., electron transport chain; tca, TCA cycle; fa, fatty acid metabolism. Further information for each sample is provided in Table 1 and Hurwitz et al. [21]. [file gb-2013-14-11-r123-S6.docx]

**Table S4.**  **Sequencing-effort weighted read abundance in viral metagenomes for genes shown in Table S3**. Column headers are: gly=glycolysis, edp=Entner–Doudoroff pathway, ppp=pentose phosphate pathway, nuc=dNTP biosynthesis, 3hp=3-hydroxypropionyl bicycle, ps=photosynthesis, etc=electron transport chain, tca=TCA cycle, fa=fatty acid metabolism. Metadata for each sample are further described in Hurwitz and Sullivan [21].

|  | gly | gly | gly | gly | gly | gly | gly | gly | gly | gly | gly |
| --- | --- | --- | --- | --- | --- | --- | --- | --- | --- | --- | --- |
| **Sample** | ***glgA*** | ***glgC*** | ***pgm*** | ***pgi*** | ***manA*** | ***pfk*** | ***fba*** | ***tpi*** | ***gap*** | ***pgk*** | ***gpm*** |
| L.Sum.O.500m | 4 | 20 | 33 | 16 | 8 | 0 | 4 | 0 | 42 | 0 | 4 |
| L.Sum.O.1000m | 3 | 3 | 28 | 14 | 20 | 3 | 3 | 8 | 11 | 0 | 0 |
| L.Sum.O.2000m | 33 | 0 | 54 | 9 | 30 | 0 | 3 | 12 | 21 | 6 | 0 |
| L.Spr.C.500m | 116 | 1 | 20 | 6 | 57 | 9 | 6 | 0 | 1 | 4 | 1 |
| L.Spr.C.1300m | 52 | 0 | 4 | 2 | 29 | 0 | 11 | 0 | 0 | 0 | 0 |
| L.Spr.I.500m | 41 | 0 | 36 | 0 | 19 | 0 | 3 | 0 | 14 | 0 | 5 |
| L.Spr.I.1000m | 49 | 0 | 0 | 0 | 33 | 0 | 0 | 1 | 2 | 0 | 0 |
| L.Spr.I.2000m | 17 | 0 | 0 | 17 | 63 | 0 | 29 | 0 | 8 | 4 | 0 |
| L.Spr.O.1000m | 12 | 0 | 6 | 2 | 28 | 2 | 4 | 0 | 0 | 0 | 0 |
| L.Spr.O.2000m | 79 | 4 | 34 | 15 | 92 | 0 | 0 | 11 | 27 | 8 | 8 |
| L.Win.O.500m | 27 | 0 | 4 | 2 | 32 | 0 | 3 | 1 | 0 | 0 | 0 |
| L.Win.O.1000m | 45 | 0 | 1 | 1 | 47 | 0 | 5 | 0 | 1 | 0 | 0 |
| L.Win.O.2000m | 23 | 0 | 12 | 2 | 11 | 2 | 2 | 2 | 12 | 0 | 0 |
| M.Fall.O.1000m | 52 | 3 | 12 | 6 | 28 | 1 | 4 | 3 | 12 | 3 | 2 |
| M.Fall.O.4300m | 42 | 1 | 1 | 3 | 33 | 0 | 5 | 0 | 1 | 2 | 3 |
| L.Sum.O.10m | 2 | 0 | 0 | 0 | 20 | 1 | 3 | 0 | 5 | 0 | 0 |
| L.Spr.C.10m | 20 | 0 | 0 | 0 | 150 | 0 | 32 | 0 | 4 | 0 | 0 |
| L.Spr.I.10m | 5 | 0 | 6 | 2 | 54 | 0 | 4 | 2 | 14 | 0 | 0 |
| L.Spr.O.10m | 23 | 3 | 15 | 3 | 58 | 0 | 3 | 0 | 29 | 0 | 3 |
| SFC.Spr.C.5m | 12 | 0 | 1 | 0 | 55 | 0 | 17 | 0 | 6 | 0 | 0 |
| SFD.Spr.C.5m | 7 | 0 | 2 | 1 | 68 | 0 | 29 | 0 | 9 | 0 | 1 |
| SFS.Spr.C.5m | 11 | 0 | 0 | 1 | 63 | 0 | 22 | 0 | 6 | 0 | 0 |
| STC.Spr.C.5m | 37 | 3 | 4 | 3 | 58 | 1 | 14 | 2 | 7 | 4 | 2 |
| GD.Spr.C.8m | 15 | 2 | 8 | 6 | 40 | 0 | 14 | 0 | 14 | 0 | 2 |
| GF.Spr.C.9m | 27 | 5 | 21 | 0 | 51 | 0 | 56 | 0 | 3 | 0 | 0 |
| L.Win.O.10m | 9 | 0 | 1 | 1 | 20 | 0 | 3 | 1 | 1 | 0 | 0 |
| M.Fall.C.10m | 25 | 0 | 1 | 0 | 36 | 0 | 20 | 1 | 2 | 0 | 1 |
| M.Fall.I.10m | 17 | 0 | 1 | 0 | 34 | 0 | 6 | 1 | 1 | 1 | 0 |
| M.Fall.O.10m | 12 | 0 | 0 | 1 | 20 | 0 | 7 | 1 | 2 | 0 | 1 |
| M.Fall.O.105m | 48 | 0 | 1 | 0 | 89 | 0 | 5 | 0 | 1 | 1 | 0 |
|  |  |  |  |  |  |  |  |  |  |  |  |
| Continued… |  |  |  |  |  |  |  |  |  |  |  |
|  | gly | gly | gly | gly | gly | edp | ppp | nuc | ppp | ppp | ppp |
| **Sample** | ***eno*** | ***ppsA*** | ***pyk*** | ***ppc*** | ***pckA*** | ***gnd*** | ***rpi*** | ***prs*** | ***rpe*** | ***tkt*** | ***tal*** |
| L.Sum.O.500m | 4 | 4 | 29 | 0 | 4 | 8 | 16 | 4 | 8 | 4 | 0 |
| L.Sum.O.1000m | 8 | 3 | 0 | 3 | 0 | 12 | 8 | 8 | 0 | 11 | 0 |
| L.Sum.O.2000m | 30 | 0 | 3 | 15 | 9 | 33 | 6 | 0 | 6 | 27 | 6 |
| L.Spr.C.500m | 3 | 4 | 10 | 3 | 29 | 19 | 14 | 0 | 17 | 42 | 5 |
| L.Spr.C.1300m | 7 | 16 | 0 | 0 | 0 | 11 | 2 | 9 | 0 | 16 | 5 |
| L.Spr.I.500m | 8 | 11 | 5 | 3 | 0 | 26 | 19 | 0 | 0 | 38 | 3 |
| L.Spr.I.1000m | 0 | 6 | 0 | 0 | 2 | 7 | 0 | 1 | 5 | 19 | 0 |
| L.Spr.I.2000m | 4 | 0 | 0 | 0 | 4 | 55 | 8 | 30 | 0 | 25 | 25 |
| L.Spr.O.1000m | 2 | 0 | 0 | 0 | 0 | 2 | 6 | 2 | 0 | 4 | 4 |
| L.Spr.O.2000m | 4 | 8 | 15 | 11 | 30 | 27 | 45 | 15 | 0 | 39 | 19 |
| L.Win.O.500m | 0 | 2 | 0 | 0 | 6 | 10 | 4 | 1 | 2 | 16 | 0 |
| L.Win.O.1000m | 0 | 0 | 0 | 0 | 0 | 1 | 6 | 0 | 1 | 8 | 1 |
| L.Win.O.2000m | 3 | 0 | 3 | 0 | 5 | 9 | 10 | 5 | 2 | 16 | 0 |
| M.Fall.O.1000m | 10 | 12 | 4 | 7 | 5 | 12 | 10 | 4 | 5 | 41 | 3 |
| M.Fall.O.4300m | 0 | 2 | 1 | 0 | 1 | 15 | 2 | 0 | 1 | 15 | 0 |
| L.Sum.O.10m | 0 | 0 | 0 | 0 | 0 | 162 | 25 | 5 | 0 | 0 | 0 |
| L.Spr.C.10m | 0 | 0 | 4 | 0 | 4 | 221 | 25 | 52 | 0 | 0 | 4 |
| L.Spr.I.10m | 2 | 2 | 0 | 0 | 2 | 36 | 70 | 2 | 0 | 24 | 5 |
| L.Spr.O.10m | 14 | 3 | 6 | 0 | 0 | 132 | 89 | 3 | 0 | 38 | 43 |
| SFC.Spr.C.5m | 0 | 0 | 0 | 0 | 3 | 72 | 20 | 18 | 0 | 5 | 21 |
| SFD.Spr.C.5m | 1 | 0 | 1 | 0 | 4 | 92 | 29 | 27 | 3 | 12 | 26 |
| SFS.Spr.C.5m | 0 | 0 | 0 | 0 | 6 | 82 | 29 | 31 | 1 | 8 | 16 |
| STC.Spr.C.5m | 3 | 2 | 4 | 2 | 4 | 51 | 25 | 22 | 5 | 21 | 24 |
| GD.Spr.C.8m | 2 | 10 | 4 | 2 | 4 | 127 | 8 | 2 | 2 | 24 | 54 |
| GF.Spr.C.9m | 5 | 13 | 5 | 3 | 16 | 66 | 5 | 11 | 3 | 11 | 37 |
| L.Win.O.10m | 1 | 0 | 0 | 0 | 0 | 108 | 6 | 1 | 0 | 2 | 4 |
| M.Fall.C.10m | 0 | 1 | 2 | 0 | 1 | 23 | 17 | 15 | 1 | 7 | 7 |
| M.Fall.I.10m | 1 | 0 | 1 | 1 | 2 | 60 | 12 | 1 | 1 | 14 | 22 |
| M.Fall.O.10m | 0 | 2 | 1 | 0 | 1 | 12 | 5 | 0 | 0 | 3 | 16 |
| M.Fall.O.105m | 0 | 1 | 0 | 0 | 1 | 36 | 13 | 0 | 8 | 3 | 205 |
|  |  |  |  |  |  |  |  |  |  |  |  |
| Continued… |  |  |  |  |  |  |  |  |  |  |  |
|  | edp | edp | edp | edp | 3hp | 3hp | 3hp | etc | etc | etc | etc |
| **Sample** | ***zwf*** | ***pgl*** | ***edd*** | ***eda*** | ***PCC*** | ***MCE*** | ***MCM*** | ***Cmplx I*** | ***Cmplx III*** | ***Cmplx IV*** | ***Cmplx V*** |
| L.Sum.O.500m | 0 | 0 | 12 | 8 | 4 | 0 | 8 | 64 | 4 | 50 | 50 |
| L.Sum.O.1000m | 0 | 0 | 14 | 6 | 28 | 0 | 0 | 110 | 31 | 65 | 37 |
| L.Sum.O.2000m | 3 | 0 | 15 | 15 | 24 | 3 | 12 | 45 | 12 | 54 | 45 |
| L.Spr.C.500m | 2 | 3 | 12 | 9 | 11 | 9 | 1 | 60 | 0 | 32 | 5 |
| L.Spr.C.1300m | 0 | 0 | 7 | 7 | 0 | 0 | 2 | 27 | 2 | 26 | 6 |
| L.Spr.I.500m | 0 | 0 | 3 | 11 | 3 | 0 | 11 | 52 | 22 | 19 | 13 |
| L.Spr.I.1000m | 0 | 0 | 0 | 0 | 0 | 4 | 0 | 6 | 0 | 8 | 0 |
| L.Spr.I.2000m | 8 | 0 | 34 | 0 | 16 | 0 | 0 | 41 | 13 | 17 | 17 |
| L.Spr.O.1000m | 0 | 0 | 6 | 2 | 2 | 0 | 8 | 10 | 0 | 2 | 8 |
| L.Spr.O.2000m | 8 | 0 | 23 | 4 | 34 | 0 | 34 | 180 | 27 | 57 | 49 |
| L.Win.O.500m | 1 | 0 | 1 | 1 | 8 | 1 | 0 | 4 | 0 | 13 | 5 |
| L.Win.O.1000m | 0 | 0 | 0 | 0 | 0 | 0 | 0 | 5 | 0 | 13 | 4 |
| L.Win.O.2000m | 2 | 0 | 2 | 3 | 7 | 0 | 3 | 17 | 13 | 5 | 10 |
| M.Fall.O.1000m | 3 | 1 | 14 | 3 | 9 | 3 | 4 | 43 | 4 | 31 | 18 |
| M.Fall.O.4300m | 3 | 1 | 3 | 0 | 6 | 1 | 3 | 8 | 4 | 8 | 7 |
| L.Sum.O.10m | 0 | 0 | 0 | 0 | 2 | 0 | 0 | 0 | 13 | 3 | 0 |
| L.Spr.C.10m | 0 | 0 | 0 | 0 | 2 | 0 | 0 | 26 | 4 | 2 | 0 |
| L.Spr.I.10m | 0 | 0 | 12 | 0 | 5 | 0 | 12 | 27 | 4 | 25 | 4 |
| L.Spr.O.10m | 3 | 0 | 17 | 22 | 15 | 0 | 9 | 105 | 43 | 32 | 32 |
| SFC.Spr.C.5m | 0 | 0 | 1 | 0 | 0 | 0 | 0 | 2 | 4 | 1 | 0 |
| SFD.Spr.C.5m | 0 | 0 | 2 | 1 | 3 | 0 | 1 | 15 | 5 | 5 | 5 |
| SFS.Spr.C.5m | 0 | 0 | 0 | 0 | 1 | 0 | 0 | 6 | 7 | 1 | 0 |
| STC.Spr.C.5m | 2 | 0 | 7 | 5 | 6 | 0 | 5 | 25 | 4 | 14 | 14 |
| GD.Spr.C.8m | 87 | 0 | 16 | 6 | 4 | 0 | 4 | 22 | 4 | 26 | 24 |
| GF.Spr.C.9m | 35 | 0 | 13 | 19 | 0 | 0 | 0 | 46 | 3 | 52 | 11 |
| L.Win.O.10m | 1 | 0 | 1 | 0 | 0 | 2 | 0 | 8 | 3 | 3 | 0 |
| M.Fall.C.10m | 0 | 1 | 1 | 0 | 2 | 0 | 0 | 6 | 3 | 5 | 0 |
| M.Fall.I.10m | 1 | 0 | 1 | 0 | 1 | 0 | 1 | 4 | 8 | 5 | 2 |
| M.Fall.O.10m | 0 | 0 | 1 | 1 | 1 | 0 | 2 | 5 | 7 | 4 | 4 |
| M.Fall.O.105m | 0 | 0 | 2 | 0 | 2 | 3 | 1 | 5 | 3 | 4 | 0 |
|  |  |  |  |  |  |  |  |  |  |  |  |
| Continued… |  |  |  |  |  |  |  |  |  |  |  |
|  | tca | tca | tca | tca | tca | tca | tca | tca | tca/etc | tca | tca |
| **Sample** | ***aceEF*** | ***gltA*** | ***acn*** | ***icd*** | ***aceA*** | ***sucAB*** | ***aceB*** | ***sucCD*** | ***sdh*** | ***fum*** | ***mdh*** |
| L.Sum.O.500m | 12 | 4 | 78 | 33 | 8 | 20 | 8 | 4 | 37 | 8 | 0 |
| L.Sum.O.1000m | 9 | 8 | 14 | 3 | 6 | 3 | 0 | 11 | 36 | 14 | 14 |
| L.Sum.O.2000m | 42 | 12 | 42 | 12 | 3 | 18 | 3 | 24 | 63 | 3 | 18 |
| L.Spr.C.500m | 10 | 3 | 19 | 4 | 63 | 5 | 30 | 3 | 17 | 9 | 5 |
| L.Spr.C.1300m | 4 | 7 | 6 | 0 | 32 | 0 | 7 | 0 | 14 | 7 | 2 |
| L.Spr.I.500m | 11 | 11 | 21 | 5 | 25 | 19 | 8 | 8 | 10 | 8 | 5 |
| L.Spr.I.1000m | 0 | 4 | 0 | 0 | 45 | 0 | 13 | 0 | 2 | 1 | 2 |
| L.Spr.I.2000m | 21 | 0 | 8 | 13 | 0 | 25 | 0 | 4 | 4 | 0 | 0 |
| L.Spr.O.1000m | 2 | 0 | 6 | 0 | 26 | 2 | 4 | 6 | 6 | 0 | 6 |
| L.Spr.O.2000m | 87 | 8 | 35 | 15 | 15 | 144 | 0 | 11 | 23 | 34 | 8 |
| L.Win.O.500m | 3 | 1 | 3 | 0 | 6 | 5 | 4 | 2 | 2 | 0 | 0 |
| L.Win.O.1000m | 0 | 2 | 0 | 0 | 15 | 1 | 8 | 0 | 3 | 0 | 1 |
| L.Win.O.2000m | 5 | 0 | 2 | 0 | 6 | 8 | 3 | 2 | 7 | 10 | 0 |
| M.Fall.O.1000m | 15 | 5 | 31 | 7 | 27 | 9 | 16 | 4 | 16 | 6 | 3 |
| M.Fall.O.4300m | 3 | 0 | 5 | 0 | 4 | 4 | 1 | 0 | 4 | 1 | 0 |
| L.Sum.O.10m | 0 | 0 | 3 | 0 | 16 | 2 | 0 | 0 | 6 | 0 | 0 |
| L.Spr.C.10m | 0 | 0 | 2 | 0 | 13 | 0 | 0 | 0 | 0 | 4 | 0 |
| L.Spr.I.10m | 7 | 10 | 19 | 2 | 6 | 7 | 4 | 0 | 11 | 7 | 2 |
| L.Spr.O.10m | 31 | 3 | 15 | 9 | 18 | 9 | 3 | 3 | 40 | 0 | 11 |
| SFC.Spr.C.5m | 0 | 1 | 1 | 0 | 1 | 0 | 0 | 0 | 0 | 0 | 0 |
| SFD.Spr.C.5m | 3 | 2 | 4 | 1 | 4 | 1 | 2 | 1 | 3 | 1 | 0 |
| SFS.Spr.C.5m | 0 | 0 | 1 | 0 | 4 | 0 | 0 | 0 | 1 | 0 | 0 |
| STC.Spr.C.5m | 8 | 2 | 13 | 2 | 1 | 3 | 2 | 5 | 13 | 4 | 1 |
| GD.Spr.C.8m | 14 | 10 | 23 | 2 | 10 | 6 | 6 | 0 | 4 | 2 | 0 |
| GF.Spr.C.9m | 29 | 0 | 14 | 0 | 13 | 8 | 8 | 11 | 14 | 5 | 0 |
| L.Win.O.10m | 0 | 0 | 1 | 0 | 14 | 1 | 0 | 1 | 2 | 0 | 1 |
| M.Fall.C.10m | 1 | 0 | 2 | 1 | 5 | 0 | 2 | 1 | 2 | 0 | 1 |
| M.Fall.I.10m | 1 | 0 | 3 | 0 | 2 | 2 | 0 | 0 | 1 | 0 | 0 |
| M.Fall.O.10m | 2 | 2 | 5 | 0 | 1 | 24 | 1 | 0 | 2 | 2 | 0 |
| M.Fall.O.105m | 1 | 0 | 0 | 0 | 2 | 6 | 0 | 0 | 1 | 0 | 0 |
| Continued… |  |  |  |  |  |  |  |  |  |  |  |

|  | fa | fa | fa | ps | ps |
| --- | --- | --- | --- | --- | --- |
| **Sample** | **ACC** | **fadB** | **fadl** | **psbA** | **psbD** |
| L.Sum.O.500m | 0 | 4 | 62 | 37 | 8 |
| L.Sum.O.1000m | 28 | 0 | 43 | 6 | 17 |
| L.Sum.O.2000m | 24 | 9 | 66 | 78 | 3 |
| L.Spr.C.500m | 8 | 5 | 54 | 28 | 24 |
| L.Spr.C.1300m | 0 | 2 | 9 | 5 | 4 |
| L.Spr.I.500m | 14 | 0 | 43 | 73 | 38 |
| L.Spr.I.1000m | 0 | 1 | 8 | 8 | 2 |
| L.Spr.I.2000m | 21 | 0 | 25 | 245 | 54 |
| L.Spr.O.1000m | 0 | 4 | 10 | 6 | 4 |
| L.Spr.O.2000m | 15 | 4 | 76 | 64 | 8 |
| L.Win.O.500m | 1 | 1 | 10 | 3 | 0 |
| L.Win.O.1000m | 0 | 1 | 6 | 4 | 0 |
| L.Win.O.2000m | 10 | 0 | 14 | 16 | 11 |
| M.Fall.O.1000m | 13 | 6 | 48 | 6 | 0 |
| M.Fall.O.4300m | 7 | 1 | 17 | 25 | 12 |
| L.Sum.O.10m | 0 | 0 | 2 | 25 | 6 |
| L.Spr.C.10m | 0 | 0 | 7 | 162 | 55 |
| L.Spr.I.10m | 7 | 0 | 47 | 97 | 32 |
| L.Spr.O.10m | 17 | 29 | 15 | 175 | 247 |
| SFC.Spr.C.5m | 1 | 1 | 3 | 171 | 113 |
| SFD.Spr.C.5m | 4 | 0 | 12 | 366 | 214 |
| SFS.Spr.C.5m | 0 | 0 | 4 | 301 | 194 |
| STC.Spr.C.5m | 6 | 1 | 46 | 95 | 66 |
| GD.Spr.C.8m | 13 | 10 | 23 | 657 | 99 |
| GF.Spr.C.9m | 5 | 16 | 29 | 458 | 67 |
| L.Win.O.10m | 0 | 1 | 0 | 33 | 15 |
| M.Fall.C.10m | 0 | 2 | 7 | 35 | 11 |
| M.Fall.I.10m | 0 | 1 | 7 | 118 | 31 |
| M.Fall.O.10m | 0 | 0 | 9 | 87 | 24 |
| M.Fall.O.105m | 0 | 1 | 9 | 599 | 64 |

|  |  |  |  |  |  |  |  |  |  |  |
| --- | --- | --- | --- | --- | --- | --- | --- | --- | --- | --- |
|  |  |  |  |  |  |  |  |  |  |  |
